# Impact of material combinations and removal and insertion cycles on the retention force of telescopic systems

**DOI:** 10.1007/s00784-023-05027-w

**Published:** 2023-04-22

**Authors:** Danka Micovic Soldatovic, Maximiliane Bitter, John Meinen, Karin Christine Huth, Anja Liebermann, Bogna Stawarczyk

**Affiliations:** 1 Department of Prosthetic Dentistry, University Hospital, LMU Munich, Goethestrasse 70, 80336 Munich, Germany; 2 Department of Conservative Dentistry and Periodontology, University Hospital, LMU Munich, Goethestrasse 70, 80336 Munich, Germany; 3 Polyclinic of Prosthetic Dentistry, Faculty of Medicine and University Hospital Cologne, University of Cologne, Kerpener Strasse 32, 50931 Cologne, Germany

**Keywords:** Double-crown system, PEEK, PEKK, Zirconia, Artificial aging, Retention force measurements

## Abstract

**Objectives:**

A variety of dental materials are available for the fabrication of telescopic crowns. The aim was to investigate the impact of material combinations and removal and insertion cycles on their retention forces.

**Materials and methods:**

CAD/CAM-fabricated cobalt–chromium–molybdenum (CoCr) and zirconia (ZrO_2_) primary crowns were combined with polyetheretherketone (PEEK), polyetherketoneketone (PEKK), CoCr, and ZrO_2_ secondary crowns (four combinations included PEEK/PEKK secondary crowns in a thickness of 0.5 mm bonded to the CoCr tertiary construction), resulting in 12 different material combinations: CoCr–PEEK; CoCr–PEKK; CoCr–ZrO_2_; CoCr–CoCr; CoCr–PEEK 0.5; CoCr–PEKK 0.5; ZrO_2_–PEEK; ZrO_2_–PEKK; ZrO_2_–ZrO_2_, ZrO_2_–CoCr; ZrO_2_–PEEK 0.5; and ZrO_2_–PEKK 0.5 (*n* = 15 pairings per material combination). Pull-off tests were performed with a universal testing machine initially and after 500, 5000, and 10,000 removal and insertion cycles in a mastication simulator. Descriptive statistics with the Kolmogorov–Smirnov, Kruskal–Wallis, and Mann–Whitney *U* tests were computed (*α* = 0.05).

**Results:**

The tested parameters, material combination, and removal and insertion cycles had significant impact on the retention force values (*p* < 0.001). An increase in removal and insertion cycles was associated with a decrease in retention forces within CoCr and ZrO_2_ secondary crowns, regardless of the primary crown material. In contrast, PEEK and PEKK secondary crowns presented higher retention load values after 10,000 cycles than initially.

**Conclusion:**

Different material combinations behaved differently after simulated removal and insertion regimens. This difference should be considered during treatment planning.

**Clinical relevance:**

Telescopic crown systems should be made of materials with predictable retention forces that do not deteriorate with time. The implementation of new materials and technologies facilitates reproducibility and time-saving fabrication.

## Introduction

Telescopic crown-retained removable partial dentures (RPDs) provide a suitable treatment option for partially edentulous patients with multiple missing teeth or in combination with extended edentulous ridges where fixed dental restorations are not indicated. These RPDs could be tooth-supported or tooth-implant-supported [1], depending on the individual situation. According to the clinical evaluations, telescopic crown-retained RPDs have been reported to show higher survival rates than conventional clasp-retained RPDs [2].

A telescopic crown system consists of a primary crown (fixed to abutment tooth or implant) and a secondary crown which is a part of the denture [3]. The contact surfaces of the primary and secondary crowns can be made almost parallel (taper angle very close to 0°). Another option is the provision of conical crowns with a recommended convergences angle of 2 to 6°. Taper, height of contact surfaces, and materials directly influence the retention values of telescopic crown systems [4–6].

In the past, precious alloys (mainly gold) were used for manufacturing primary and secondary crowns. Gold alloys have excellent biocompatibility, can be easily processed by a dental laboratory technician, and allow the required retention forces to be adjusted [7]. Due to the high cost of precious alloys, non-precious alloys, for example, cobalt–chromium–molybdenum (CoCr), have been used for this application. CoCr crowns can be conventionally manufactured using the lost-wax casting technique, but, due to the higher modulus of elasticity (≈ 210 GPa) in comparison with gold alloys (≈ 150 GPa), the process of fabrication and adaptation is more difficult and error-sensitive. Recently, with the help of computer-aided design and computer-aided manufacturing (CAD/CAM), CoCr telescopic crowns can be milled or even 3D printed. Although this alloy exhibited satisfactory characteristics regarding retentive behavior [7], precise fitting, and flexural strength [8], its biocompatibility is questionable. The combination of CoCr alloy with other metal alloys in wet oral conditions could lead to the dissolution of metal ions and galvanic corrosion [9]. The current trend towards non-metallic restorations and increased esthetic demands, as well as the high number of allergy-prone patients, has led to the introduction of new prosthodontic materials.

Advanced CAD/CAM dental technologies led to rapid and cost-effective production, overcoming the problems of conventional casting [5]. CAD/CAM technology also enabled the use of improved ceramic and polymer-based materials, including zirconia and polyaryletherketone (PAEK).

Zirconia has excellent esthetic and mechanical properties, biocompatibility, and long-term stability, all of which make it suitable for implants, abutments, frameworks for fixed dental restorations, and monolithic fixed prostheses [10, 11]. Zirconia has been reported to be a suitable primary crown material [12], especially in combination with electroformed gold secondary crowns [13]. However, the combination of zirconia with non-precious alloy secondary crowns has been reported to cause significant wear and loss of friction [5]. Studies testing zirconia as a secondary crown material have reported contradictory findings [14], and further studies are necessary for more accurate results and for providing reliable recommendations.

Polymer-based dental materials, including PAEK, have become popular with CAD/CAM systems. The PAEK family consists of a variety of high-performance thermoplastic polymers which differ in the number of functional ether- or keto-groups. These include polyetheretherketone (PEEK), polyetherketoneketone (PEKK) [15], and the recently developed high-performance aryl-ketone polymer (AKP) [16]. Because of their slightly different composition, their properties and thus also the indication area differ [17]. PEEK has been previously tested as part of a telescopic crown system in a few in vitro investigations and was reported as a suitable material for this indication [3, 18]. However, the authors are only aware of a case report [19] and an in vitro study [20] that examined PEKK as a telescopic crown material, reporting promising results for this indication. Excellent mechanical properties, biocompatibility, chemical stability, low plaque adhesion, and a broad range of processing options (milling, pressing, 3D-prinitng) make PAEK materials attractive for wider implementation in prosthetic dentistry [21].

Another traditional approach is the electroplating of secondary crowns. In this procedure, gold ions are deposited under electric current to produce accurately fitting gold copings [22]. Crowns made in this way do not need to be adjusted, as do conventionally cast secondary crowns, but are intraorally bonded to the tertiary structure, ensuring a passive fit of the restoration with excellent stress distribution. However, the technically demanding and time-consuming fabrication process leads to an expensive dental restoration, and whether milled PEEK or PEKK copings could be an affordable alternative to gold is unclear. Milled PEEK or PEKK might overcome the drawbacks of the electroplating technique by facilitating the fabrication of reproducible copings and the passive fit of the tertiary structure. If the retention force changes over time, a PEEK or PEKK coping could be easily replaced without fabricating a completely new restoration.

The goals of this investigation were to examine and compare the behavior of the retention forces of different material pairings, simulating function with artificial aging (removal and insertion cycles). The null hypotheses were that material combinations would not impact retention force on one aging level and that thermomechanical aging would not impact the retention force values of one material combination.

## Materials and methods

The retention load of telescopic crowns made of different materials was investigated in the present investigation (Table 1). Cobalt–chromium–molybdenum (CoCr) alloy and zirconia (ZrO_2_) were used as primary crown materials. For each material, 15 secondary crowns were produced using polyetheretherketone (PEEK), polyetherketoneketone (PEKK), CoCr alloy, and ZrO_2_.

**Table 1:** Summary of used materials

	Material	Manufacturer	LOT number
• Primary crown	Cobalt–chromium–molybdenum (CoCr), Ceramill sintron	Amann Girrbach	1303045, 1700661
	Zirconia (ZrO_2_), Ceramill ZI	Amann Girrbach	1303002
• Secondary crown	Polyetheretherketone (PEEK), BioHPP	bredent	504894, 496211, 495767, 486101
	Polyetherketoneketone (PEKK), Pekkton	Cendres+Métaux	204280, 211144, 211145
	Cobalt-chromium (CoCr)	a.m.	a.m.
	Zirconia (ZrO_2_)	a.m.	a.m.
• Tertiary crown	Cobalt-chromium (CoCr)	a.m.	a.m.
• Bonding	AGC Cem Automix System	C. Hafner	220868
	visio.link	bredent	193211
	MKZ primer	bredent	494986

**Fig. 1:**
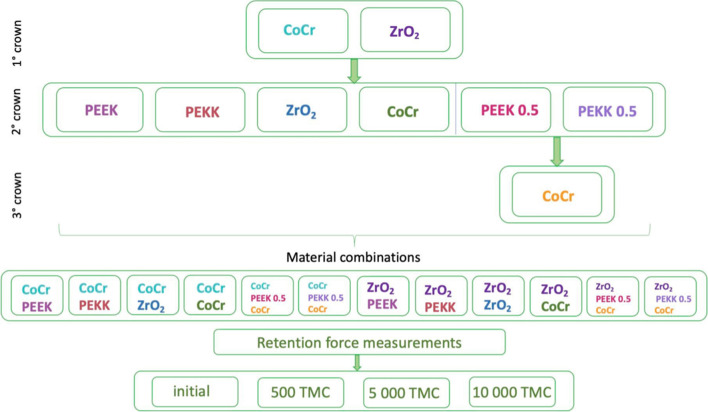
Study design

**Fig. 2:**
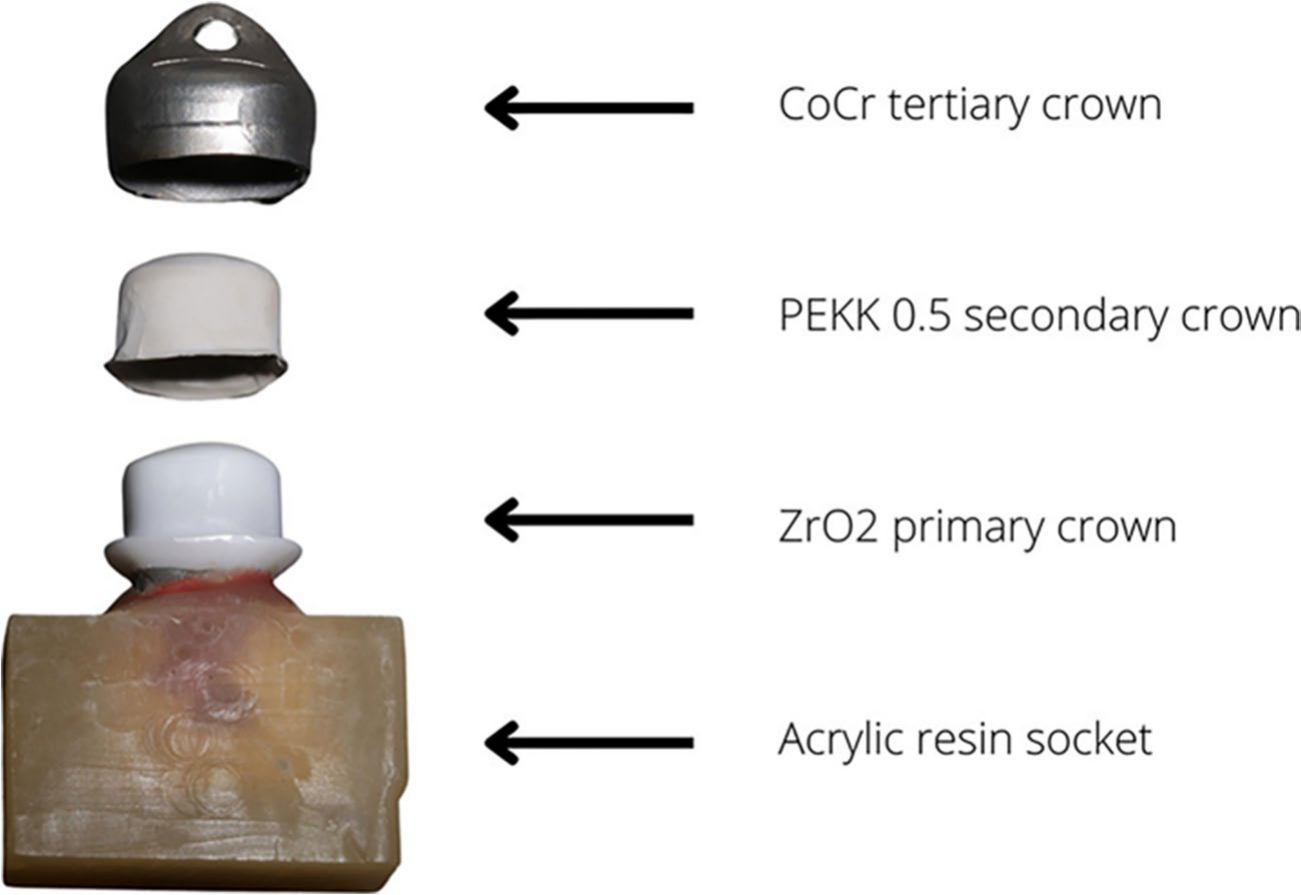
Three-element system components

Four groups were designed as three-element systems with tertiary constructions where the primary crowns were made of CoCr/ZrO_2_ and secondary PEEK/PEKK in a thickness of 0.5 mm simulating the electroplated copings and CoCr tertiary crowns (Fig. 2). This resulted in 12 groups of material pairings with 15 specimens per group (Fig. 1).

### Specimen manufacturing

#### Primary crowns

To obtain a basis for the abutments, a prepared plastic model of the maxillary first molar (26) was duplicated with a silicone mold (Adisil blau 9:1, Siladent). Thirty wax abutments were manufactured and converted into CoCr abutments (Remanium GM 800+, Dentaurum) using the conventional lost-wax technique.

Each of these abutments was scanned (Ceramill map 300, Amann Girrbach) and digitized in a CAD software program (Ceramill Mind, Amann Girrbach). Based on this, parallel primary crowns (cone angle 0°) with chamfer preparation were designed and subsequently milled from CoCr alloy (Ceramill Sintron, Amann Girrbach) and zirconia (Ceramill ZI, Amann Girrbach). The primary crowns were sintered according to the manufacturer’s instructions: CoCr crowns in a protective atmosphere (argon: 1 bar, compressed air: 1.2 bar; Ceramill Argotherm, Amann Girrbach) and zirconia crowns following the program: heat up to 1450 °C (5–10 K/min), with a dwell time of 2 h and a cooling rate of 5 K/min until room temperature.

Both types of sintered primary crowns were adhesively bonded to the abutments (RelyX Unicem 2, 3M). All bonded primary crowns were parallel mounted in acrylic resin sockets (Scandiquick, Scan-Dia) to ensure stability during pull-off tests and subjected to artificial aging. The insertion direction was defined using a turbine (W&H Perfecta 900; W&H Dentalwerk) positioned in a parallelometer (F4 basic, DeguDent) with constant water cooling. All primary crowns (*n* = 15 CoCr, *n* = 15 Zr0_2_ crowns) were high-gloss polished.

#### Secondary crowns

A total of 180 secondary crowns were manufactured using four different materials. Each primary crown was scanned (Ceramill map 300) to construct a corresponding secondary crown with a CAD software program (Ceramill Mind). All secondary crowns were constructed individually without cement spacer or block outs, with a 2-mm thickness and a ridge on the occlusal surface (provided to make a hole to perform pull-off tests and mount specimens in the mastication simulator). The same STL data were used to mill (Ceramill Motion 2) PEEK (breCAM.BioHPP, bredent) and PEKK (Pekkton, Cendres+Métaux) secondary crowns due to the similarity of materials. In order to mill CoCr (Ceramill Sintron, Amann Girrbach) and ZrO_2_ (Ceramill ZI, Amann Girrbach) secondary crowns, the parameters, like cement spacer, were adjusted so that all types of secondary crowns had at the baseline retention force of 10–15 N.

Previously used STL files were optimized to mill PEEK and PEKK secondary crowns which were used in combination with CoCr tertiary crowns. All the parameters were the same except for the thickness of the crown (reduced to 0.5 mm), and they were constructed without an occlusal ridge: PEEK 0.5 and PEKK 0.5.

This resulted in 12 different groups of material pairings (Table 1; Fig. 1).

#### Tertiary crowns

For four groups (CoCr–PEEK 0.5; CoCr–PEKK 0.5; ZrO_2_–PEEK 0.5; and ZrO_2_–PEKK 0.5) tertiary crowns were fabricated (Fig. 2). The secondary PEEK/PEKK 0.5 crown was positioned on its corresponding primary crown and then scanned and digitized. The tertiary construction (with occlusal ridge) was milled from CoCr, sintered, and high-gloss polished using polishing brushes and paste (Komet Dental; Abraso-Starglanz, bredent).

Prior to initial retention force measurements, secondary PEEK 0.5/PEKK 0.5 crowns were adhesively bonded to tertiary CoCr crowns and incubated for 24 h at 37 °C (HERAcell 150, Thermo Scientific).

The bonding procedure consisted of airborne-particle abrasion of both surfaces (PEEK/PEKK and CoCr) with 50-μm Al_2_O_3_, with a pressure of 2 bars, cleaning in an ultrasound bath, applying a thin layer of MKZ primer (bredent) on the CoCr intaglio surface, drying for 60 s, applying a thin layer of visio.link (bredent) on the outer PEEK/PEKK surface and polymerization for 90 s (bre.lux power unit, bredent), filling CoCr crown with AGC autopolymerizing compomer cement (AGC Cem Automix system, C Hafner), and pressing onto the secondary crown which had been positioned on corresponding primary crown which had been previously isolated with a thin layer of Vaseline.

The fit of every secondary crown was tested, and the initial retention force was adjusted by grinding the intaglio surface of the secondary crown to obtain 10–15 N for each pairing. After the adjustment, the intaglio surfaces were polished, and the crowns made ready for the initial retention force measurements.

### Retention force measurement and artificial aging

**Fig. 3:**
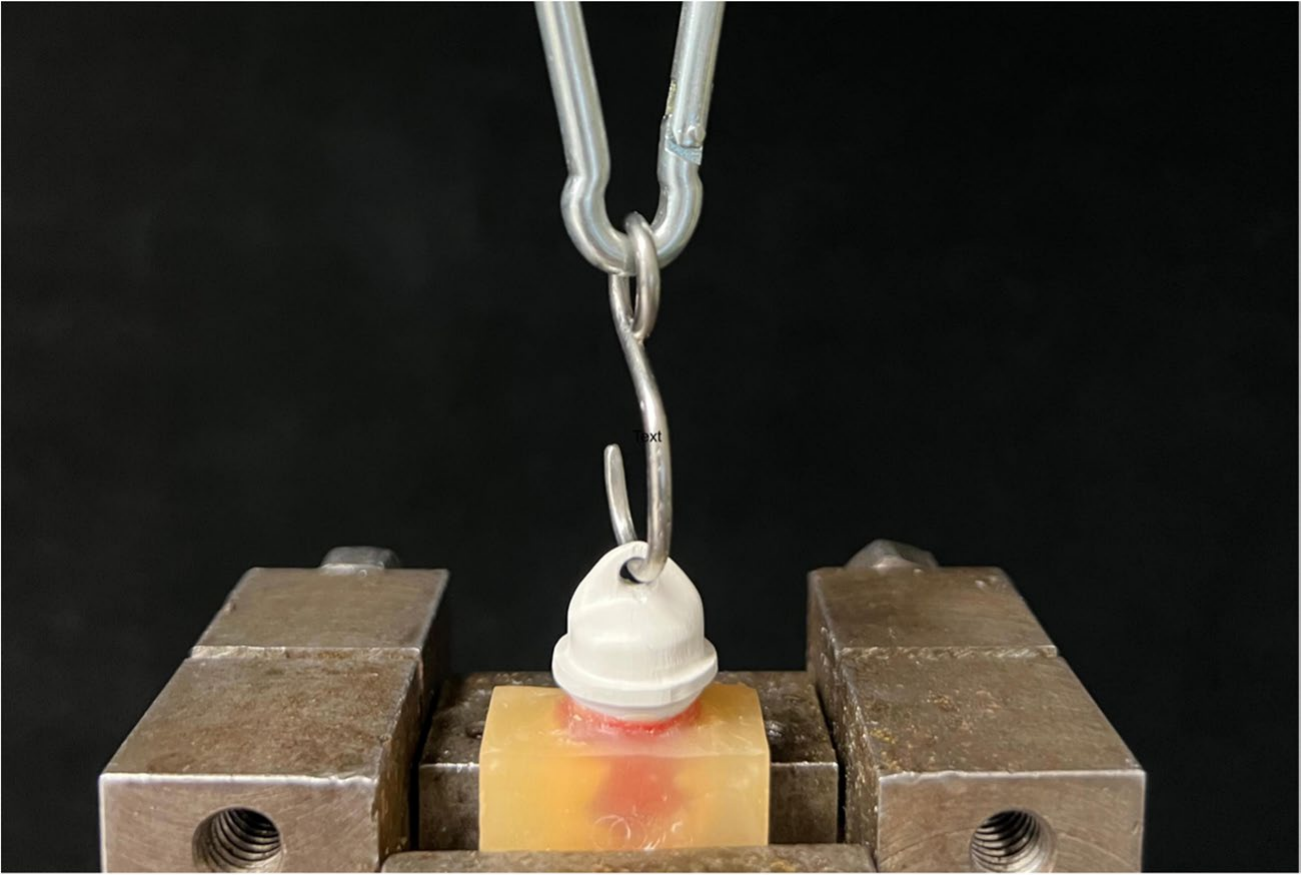
Retention force measurement setup

Retention force measurements were performed in a universal testing machine (Zwick 1445, Zwick/Roell). The primary crown on its acrylic resin base was fixed in the machine. The secondary crown was wetted with an artificial saliva spray (Glandosane, cell pharm) and fitted onto the primary crown. Using a hook through the hole in the occlusal ridge of the secondary crown, pull-off tests were done at a speed of 50 mm/min (Fig. 3). The experimental setup was already proven in several investigations [8, 13, 23, 25, 26].

According to the study design (Fig. 1), each specimen was exposed to 500, 5000, and 10,000 thermomechanical cycles in a mastication simulator (SD Mechatronic). The cycles corresponded to approximately 6 months, 5 years, and 15 years (respectively) in clinical conditions when patients remove the restoration three times per day [3, 18, 20]. A mechanical load of 50 N was applied, and the thermal cycles consisted of temperature changes between 5 and 55 °C with a 60-s dwell time.

A parallelometer was used to ensure the specimens were in the same position each time they were mounted in the mastication simulator. Acrylic resin sockets with primary crowns and secondary crowns were fixed for the antagonistic parts of the mastication simulator, securing the path of insertion during aging. Pull-off tests and loading during aging were executed in an axial direction, parallel to the insertion direction and perpendicular to the model base. After each aging interval, five pull-off tests per pairing were performed and retention force values were recorded.

### Statistical analyses

For power analysis, the results from a prior study [23] on the initial retention load of primary and secondary zirconia crowns (17.63 ± 5.16 N) were used for calculation (nQuery + nTerim, Version 3.0, Statistical Solutions). The aim of this power analysis was to determine cross-sectional differences after aging using chewing simulator between the tested secondary crowns. A sample size of 15 in each of 12 material combinations had a 95% power to detect a difference in retention load means of 8.81 N (50% reduction), assuming that the common standard deviation of retention load was 5.16 N using a two group *t*-test with a Bonferroni corrected two-sided significance level (*α* = 0.008).

The assumption of normality was tested with the Kolmogorov–Smirnov test. The descriptive statistics mean, standard deviation (SD), and 95% confidence interval (CI) were computed. The Kruskal–Wallis test was used to disclose differences in mean retention load between 12 tested material combinations. The Mann–Whitney *U* test was performed to estimate the effect of material combination and removal and insertion cycles on retention load values. A statistical software program (IBM SPSS Statistics, version 26.0.0.1, IBM Corp) was used for the analyses (*α* = 0.05).

## Results

As the measured data deviated from normal distribution (64.6%), non-parametric tests were performed (Table 2). The tested parameters, material combination, and removal and insertion cycles were shown to impact the retention force values (*p* < 0.001). The highest impact showed material combination (*η*_p_^2^ = 0.542), followed by interaction between material combination and removal and insertion cycles (*η*_p_^2^ = 0.266) and removal and insertion cycles (*η*_p_^2^ = 0.079). Descriptive statistics are summarized in Table 2. All material combinations showed differences in retention load values regardless of removal and insertion cycles (*p* < 0.001) (Table 2).

**Table 2:** Descriptive statistics. All values for retention load in Newton (N)

Material combinations			Removal and insertion cycles							
			Initial		500 TMC		5000 TMC		10,000 TMC	
1°	2°	3°	Mean ± SD	[95% CI]	Mean ± SD	[95% CI]	Mean ± SD	[95% CI]	Mean ± SD	[95% CI]
CoCr	PEEK		13.1 ± 2.9*^ef A^	[12.2; 13.8]	12.0 ± 4.2^cd A^	[10.9; 13.0]	17.3 ± 5.6^df B^	[15.8; 18.6]	20.3 ± 4.6^f C^	[19.1; 21.4]
CoCr	PEKK		11.6 ± 2.5*^cde A^	[10,9; 12,1]	10.4 ± 3.5*^c A^	[9.5; 11.3]	18.3 ± 5.2^f B^	[17.0; 19.6]	19.6 ± 5.5*^ef B^	[18.2; 21.0]
CoCr	ZrO_2_		10.4 ± 2,8*^abc D^	[9.3; 10.8]	6.1 ± 3.3^ab C^	[5.2; 6.9]	3.9 ± 2.9*^a B^	[3.1; 4.7]	2.2 ± 1.9*^a A^	[1.6; 2.7]
CoCr	CoCr		12.2 ± 2.3^def B^	[11.6; 12.8]	7.6 ± 2.7^b A^	[6.9; 8.3]	7.7 ± 3.9*^bc A^	[6.6; 8.7]	9.1 ± 4.7*^bc A^	[7.9; 10.3]
CoCr	PEEK 0.5	CoCr	13.1 ± 2.1*^ef A^	[12.6; 13.7]	12.4 ± 3.2*^cde A^	[11.6; 13.2]	16.8 ± 5.2^df B^	[15.5; 18.1]	15.8 ± 5.6^d B^	[14.4; 17.2]
CoCr	PEKK 0.5	CoCr	13.3 ± 2.0^f A^	[12.7; 13.8]	15.7 ± 3.7*^f B^	[14.7; 16.6]	18.5 ± 4.7*^f C^	[17.3; 19.7]	15.9 ± 3.9*^d B^	[14.9; 16.9]
ZrO_2_	PEEK		9.1 ± 2.2*^a B^	[8.4; 9.6]	7.4 ± 2.3*^b A^	[6.7; 8.0]	9.7 ± 2.8*^c B^	[8.9; 10.1]	11.1 ± 3.8^c C^	[10.1; 12.1]
ZrO_2_	PEKK		12.1 ± 2.6*^def A^	[11.3; 12.8]	10.8 ± 3.4*^cd A^	[9.8; 11.6]	15.0 ± 4.1*^d B^	[13.9; 16.0]	18.1 ± 7.8*^def C^	[16.1; 20.1]
ZrO_2_	ZrO_2_		9.8 ± 2.3*^ab D^	[9.1; 10.4]	4.6 ± 2.5*^a C^	[4.0; 5.3]	2.7 ± 2.8*^a B^	[2.0; 3.5]	1.6 ± 1.3*^a A^	[1.2; 2.0]
ZrO_2_	CoCr		10.9 ± 2.0^bcd B^	[10.4; 11.5]	4.8 ± 2.5*^a A^	[4.1; 5.5]	5.4 ± 4.5*^ab A^	[4.2; 6.5]	6.1 ± 5.0*^b A^	[4.9; 7.4]
ZrO_2_	PEEK 0.5	CoCr	13.0 ± 2.3^ef A^	[12.4; 13.6]	13.0 ± 3.3*^de A^	[12.1; 13.9]	15.0 ± 5.1*^d A^	[13.7; 16.2]	16.1 ± 6.4*^de B^	[14.5; 17.7]
ZrO_2_	PEKK 0.5	CoCr	13.7 ± 1.5^f A^	[13.3; 14.1]	14.3 ± 3.5^ef A^	[13.4; 15.2]	18.8 ± 5.1^f B^	[17.5; 20.1]	18.5 ± 4.3^def B^	[17.3; 19.6]

*Not normal distributed groups

^a, b, c, d, e, f^Different homogeneity material combination

^A, B, C, D^Different homogeneity removal and insertion cycles

Within initial measurements, ZrO_2_–PEEK showed lower values compared with ZrO_2_–CoCr, CoCr–PEKK, ZrO_2_–PEKK, CoCr–CoCr, ZrO_2_–PEEK 0.5, CoCr–PEEK, CoCr–PEEK 0.5, CoCr–PEKK 0.5, and ZrO_2_–PEKK 0.5 (*p* < 0.001). The highest values were found for ZrO_2_–PEKK 0.5 and CoCr–PEKK 0.5. These groups showed higher retention load values than CoCr–PEKK, ZrO_2_–CoCr, CoCr–ZrO_2_, ZrO_2_–ZrO_2_, and ZrO_2_–PEEK (*p* < 0.001).

After 500 removal and insertion cycles, ZrO_2_–ZrO_2_ and ZrO_2_–CoCr showed similar retention load values (*p* = 0.625) which were significantly lower in comparison with ZrO_2_–PEEK, CoCr–CoCr, CoCr–PEKK, ZrO_2_–PEKK, CoCr–PEEK, CoCr–PEEK 0.5, ZrO_2_–PEEK 0.5, ZrO_2_–PEKK 0.5, and CoCr–PEKK 0.5 (*p* < 0.001). The highest retention load values after 500 cycles were for the CoCr–PEKK 0.5 material combination which were similar to the ZrO_2_–PEKK 0.5 (*p* = 0.097) but differed significantly (*p* < 0.001) from all other material combinations.

After 5000 removal and insertion cycles, the lowest retention load values were for ZrO_2_–ZrO_2_ and CoCr– ZrO_2_ compared with CoCr–CoCr, ZrO_2_–PEEK, ZrO_2_–PEKK, ZrO_2_–PEEK 0.5, CoCr–PEEK 0.5, CoCr–PEEK, CoCr–PEKK, CoCr–PEKK 0.5, and ZrO_2_–PEKK 0.5 (*p* < 0.001). The highest values were measured for ZrO_2_–PEKK 0.5 which were similar to CoCr–PEKK 0.5 (*p* = 678), CoCr–PEKK (*p* = 0.453) and CoCr–PEEK (*p* = 0.067) material combinations. They differed significantly from ZrO_2_–PEEK, CoCr–CoCr, ZrO_2_–CoCr, CoCr– ZrO_2_, and ZrO_2_–ZrO_2_ (*p* < 0.001).

After 10,000 removal and insertion cycles, CoCr–PEEK exhibited the highest values, which were similar to those of CoCr–PEKK (*p* = 0.232). Between CoCr–PEKK and ZrO_2_–PEKK 0.5, there was also no significant difference (*p* = 0.519). ZrO_2_–ZrO_2_ and CoCr–ZrO_2_ had the lowest retention load values after 10,000 cycles, which was significantly different from those of all other material combinations (*p* < 0.001).

**Fig. 4:**
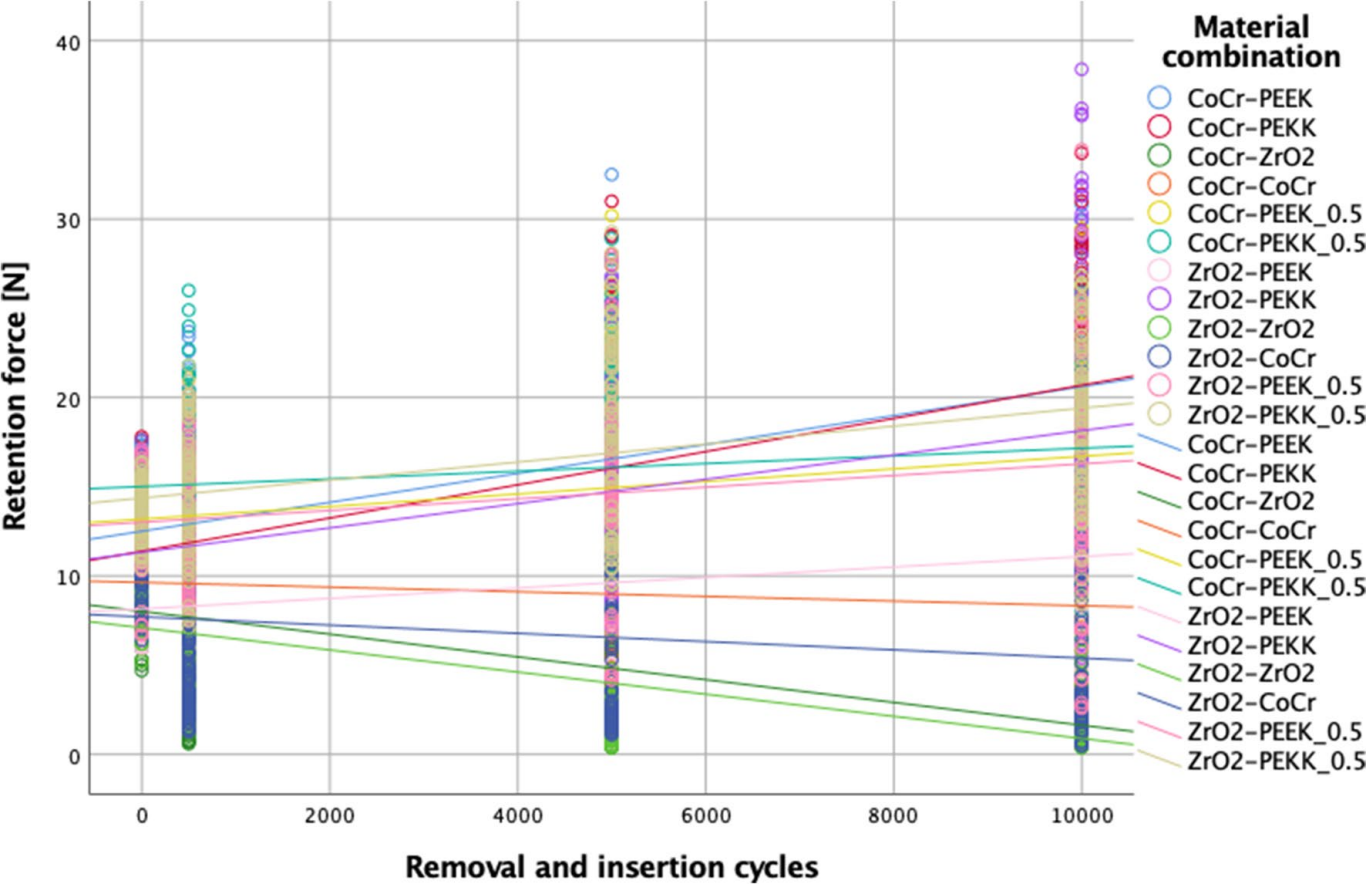
Retention load values of different material combinations measured initially and after 500, 5000, and 10,000 removal and insertion cycles

The behavior of all tested material combinations after different aging regimens is illustrated in Fig. 4.

**Fig. 5:**
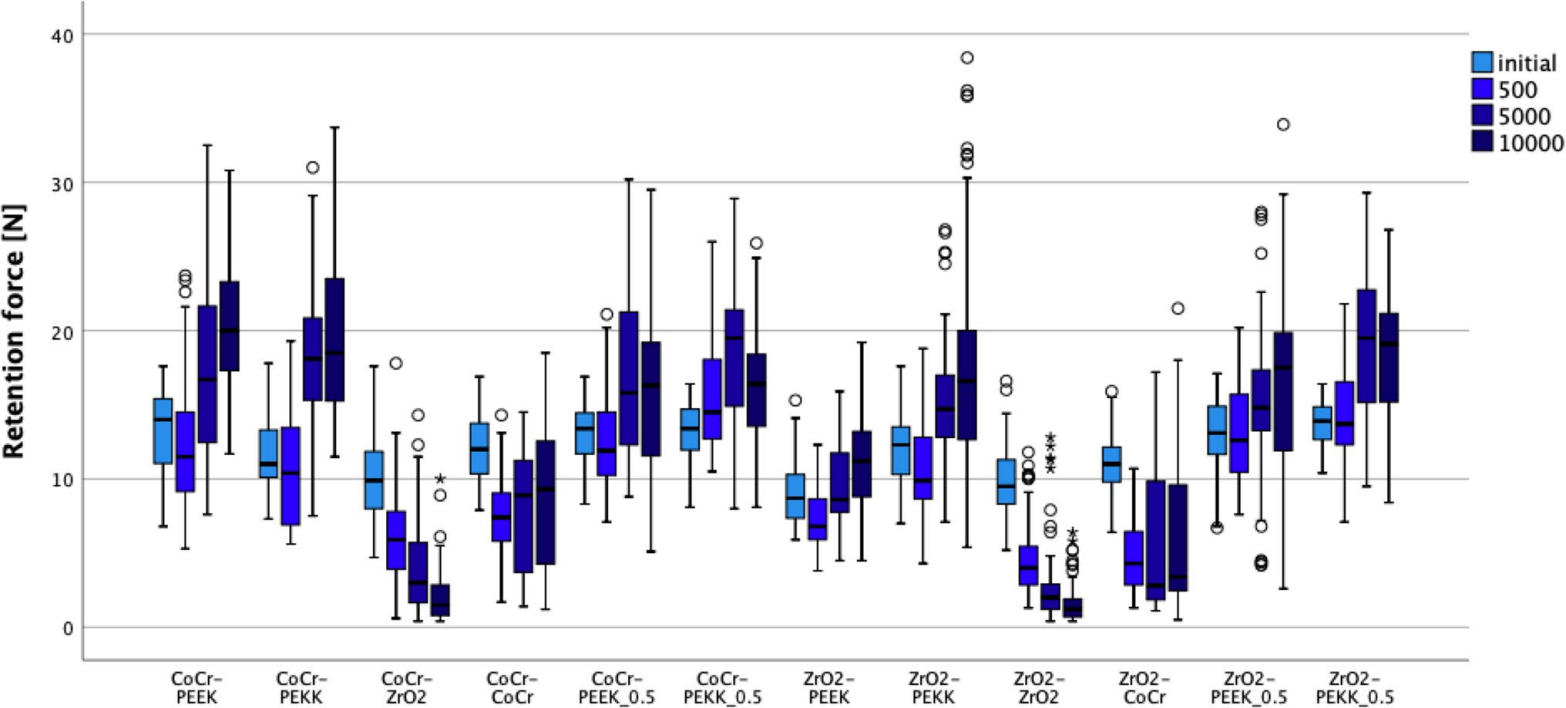
Performance of different material pairings initial and after 500, 5000, and 10,000 removal and insertion cycles

An increase in removal and insertion cycles showed differences in retention load values independent of material combinations (*p* < 0.01) (Table 2; Fig. 5). Within CoCr–PEEK, CoCr–PEKK, CoCr–PEEK 0.5, CoCr–PEKK 0.5, ZrO_2_PEKK, and ZrO_2_PEKK 0.5 material combination, the initial retention force and after 500 cycles showed lower values than after 5000 and 10,000 cycles. In addition, within CoCr–PEEK and ZrO_2_–PEKK, material combination retention force increased between 5000 and 10,000 cycles and within CoCr–PEKK 0.5; no differences were found between 500 and 10,000 cycles. Within ZrO_2_–PEEK 0.5 material combination after 10,000 cycles, higher values were found compared with initial and 500 cycles. Within the ZrO_2_–PEEK material combination, the lowest values were found after 500 and the highest after 10,000 cycles.

In contrast, within the CoCr–ZrO_2_ and ZrO_2_–ZrO_2_ material combination, a decrease in retention load was observed with an increase in removal and insertion cycles. Within the CoCr–CoCr and ZrO_2_–CoCr material combination, initial values showed higher retention force than after removal and insertion cycles (Fig. 5).

## Discussion

This investigation examined the influence of material combinations and removal and insertion cycles on the retention load values of telescopic systems. Specimens exposed to 500, 5000, and 10,000 removal and insertion cycles in combination with temperature changes simulated a clinical lifetime of more than 15 years.

The obtained results showed that material combination as well as removal and insertion cycles significantly impacted the retention load values (Fig. 4); therefore, both null hypotheses were rejected. The retention forces declined constantly and significantly when zirconia secondary crowns were tested on both types of primary crowns. Similar results presented for CoCr secondary crowns where initial retention forces were higher than after thermomechanical cycling. This retention reduction might be explained by wear from the friction between the contacting surfaces during the removal and insertion cycles.

Within material combinations which included PEEK or PEKK, an increase of retention forces was observed between baseline and 10,000 cycles. This increase could be explained by the elasticity and adaptability as well as by the reduced wear of polymer materials and was consistent with a previous investigation [24] which reported that a PEEK–PEEK combination remained constant during aging but that PEEK secondary crowns in combination with ZrO_2_ primary crowns exhibited an increase in retention force. On the other hand, the increase of retentive forces may be the consequence of interfacial wear or deformation leading to settling of the components of telescopic system. This can result in tight fit beyond that which is clinically acceptable. Hence, the increase of retention forces cannot be always understood as an advantage, and further investigations, including SEM imaging, shall provide us with more information. The retention force of PEEK crowns was also raised by increasing the number of pairings tested simultaneously.

The authors are unaware of a previous study that tested PEEK or PEKK secondary crowns as part of a three-system prosthesis. The hypothesis was to determine whether PEEK/PEKK crowns in a thickness of 0.5 mm could replace gold copings as part of a three-system prosthesis to reduce costs, avoid technically demanding procedures, and achieve reproducibility and completely metal-free restorations. According to the obtained results, retention forces measured within the material combinations, including PEEK/PEKK_0.5, showed comparable behavior with that of the PEEK and PEKK secondary crowns, indicating that an increase in retention force values was observed with increased removal and insertion cycles.

The high retention load values obtained during this experiment might be explained by the relatively large friction surface of 175 mm^2^. The high retention load values of PEEK/PEKK crowns may be a result of the oversized contact surfaces and because a dimensional reduction of the crowns would decrease the retention load values, making them more clinically relevant. However, the enlarged contact area was used to ensure increased retention forces to obtain comparable values. In addition, the results obtained were comparable with those of previous investigations with a similar experimental design [8, 23, 25, 26].

All secondary crowns were produced by milling, and the results were consistent with those of an investigation that stated that the digital workflow might provide predictable retention forces and be a suitable alternative to the conventional workflow [5]. Retention force measurements were performed under wet conditions using artificial saliva, whereas distilled water was used for removal and insertion cycles. According to previous investigations, moist conditions are important for generating hydraulic forces between primary and secondary crown (like saliva in the clinical situation), and no differences were found between artificial saliva and distilled water [3].

Artificial saliva and thermomechanical loading in a mastication simulator, with removal and insertion cycles as well as temperature changes, were attempts to simulate oral conditions. However, limitations of this investigation included the in vitro study design, oversized specimens, and retention load measurements that were always performed on only one material pairing, which does not correspond to the clinical situation, as telescopic prostheses consist of at least two or more telescopic crowns. Further investigations should use different tapers of telescopic crowns, increase the number of telescopic systems acting simultaneously, and use specimens with tooth like dimensions to improve recommendations for clinical application.

## Conclusions

Within the limitations of this investigation, it was concluded that different material combinations have different retention forces, which should be considered during treatment planning. Furthermore, the simulation of approximately 15 years of clinical use resulted in a decrease in the retention forces for ZrO_2_ and CoCr secondary crowns on both types of primary crowns, while an increase in retention load values was demonstrated for PEEK and PEKK secondary crowns. This increase of retention forces should be further investigated.
